# Unveiling novel genes upregulated by both rhBMP2 and rhBMP7 during early osteoblastic transdifferentiation of C2C12 cells

**DOI:** 10.1186/1756-0500-4-370

**Published:** 2011-09-26

**Authors:** Juan C Bustos-Valenzuela, Andre Fujita, Erik Halcsik, Jose M Granjeiro, Mari C Sogayar

**Affiliations:** 1 Chemistry Institute, Department of Biochemistry, Cell and Molecular Therapy Centre (NUCEL), University of São Paulo, Avenida Prof. Lineu Prestes, 748 Bloco 9S, São Paulo, SP 05508-000, Brazil; 2 Institute of Mathematics and Statistics, Department of Computer Science, University of São Paulo, Rua do Matão 1010, São Paulo, SP, 05508-090, Brazil; 3 National Institute of Metrology, Standardization and Industrial Quality (INMETRO), Bioengineering Sector, Duque de Caxias, RJ, CEP 25250-020, Brazil

## Abstract

**Findings:**

We set out to analyse the gene expression profile of pre-osteoblastic C2C12 cells during osteodifferentiation induced by both rhBMP2 and rhBMP7 using DNA microarrays. Induced and repressed genes were intercepted, resulting in 1,318 induced genes and 704 repressed genes by both rhBMP2 and rhBMP7. We selected and validated, by RT-qPCR, 24 genes which were upregulated by rhBMP2 and rhBMP7; of these, 13 are related to transcription (*Runx2, Dlx1, Dlx2, Dlx5, Id1, Id2, Id3, Fkhr1, Osx, Hoxc8, Glis1, Glis3 *and *Cfdp1*), four are associated with cell signalling pathways (*Lrp6, Dvl1, Ecsit *and *PKCδ*) and seven are associated with the extracellular matrix (*Ltbp2, Grn, Postn, Plod1, BMP1, Htra1 *and *IGFBP-rP10*). The novel identified genes include: *Hoxc8, Glis1, Glis3, Ecsit, PKCδ, LrP6, Dvl1, Grn, BMP1, Ltbp2, Plod1, Htra1 *and *IGFBP-rP10*.

**Background:**

BMPs (bone morphogenetic proteins) are members of the TGFβ (transforming growth factor-β) super-family of proteins, which regulate growth and differentiation of different cell types in various tissues, and play a critical role in the differentiation of mesenchymal cells into osteoblasts. In particular, rhBMP2 and rhBMP7 promote osteoinduction *in vitro *and *in vivo*, and both proteins are therapeutically applied in orthopaedics and dentistry.

**Conclusion:**

Using DNA microarrays and RT-qPCR, we identified both previously known and novel genes which are upregulated by rhBMP2 and rhBMP7 during the onset of osteoblastic transdifferentiation of pre-myoblastic C2C12 cells. Subsequent studies of these genes in C2C12 and mesenchymal or pre-osteoblastic cells should reveal more details about their role during this type of cellular differentiation induced by BMP2 or BMP7. These studies are relevant to better understanding the molecular mechanisms underlying osteoblastic differentiation and bone repair.

## Background

Bone formation and fracture repair depends on the expression and action of the bone morphogenetic proteins (BMPs), which are members of the transforming growth factor beta (TGF-beta) superfamily of dimeric, disulphide-linked growth factors, comprising more than 15 related proteins. In addition to a crucial role in osteogenesis, BMPs display a myriad of roles in cell proliferation, differentiation, migration and apoptosis, in different cell types [1]. Their role is essential at early phases of development and organogenesis, such as axial embryo determination [2], as well as in limb, eye and kidney development, such that ablation of these genes results in death at very early stages of development, as observed in knock-out mice [3]. In humans, recombinant BMP2 and BMP7 have gained attention in bone repair and in non-union spinal fractures due to their capacity to stimulate the differentiation of mesenchymal stem cells from the periosteum near the lesion site after migration and proliferation induced by IL-1, IL-6, and TNF-α [4]. This feature was first observed in the 1960s through the ectopic bone formation activity induced by bone extracts [5]. However, since only a few clinical trials concerning the use of these proteins are available, questions about the amount of BMPs required for complete bone regeneration and the extent of side effects caused by their application remains unclear.

BMPs can activate osteoblastic differentiation by binding to two different surface receptor classes on the cells, namely: type I receptors or activin receptor-like kinases (Alk 2, 3 and 6) and type II receptors (BMPR2 and activin A receptors type IIA and IIB), which are constitutively active, and transfer a phosphoryl group to serine and threonine residues in type I receptors upon ligand binding. Nevertheless, this binding is specific for each type of BMP, since BMP2 displays higher affinity for the Alk2 and Alk3 receptors, whereas BMP7 has more affinity for Alk2 receptors [6]. In addition, the downstream signalling pathway depends on how these receptors are disposed. When the receptors are already dimerised (PFC; pre-formed complex) prior to BMP binding, the Smad downstream pathway is activated through the Smad proteins 1, 5 and 8, which activate Runx2, Dlx5 and Osterix (Osx). In the BMP-induced signalling complex (BISC), BMP binding leads to receptor dimerisation; the caveosome-directed pathway leads to MAPK activation, which leads to an induction of RunX2 expression and phosphorylation, thus determining osteoblastic differentiation [1]. Moreover, *in vitro *studies have shown activation of the Osx proteins in Smad knockdown cell lines, indicating independent MAPK-activated osteoblastic differentiation. On the other hand, ERK1/2 has been found to inhibit Smad and halt bone formation [7]. These findings suggest that osteoblastic differentiation is a time-dependent process, involving the activation and inhibition of different substrates and expression of different types of genes whose nature remains to be elucidated. To investigate the transcriptional events which are independently regulated by BMP2 and BMP7, we used a gene expression microarray platform containing DNA sequences corresponding to 36,000 genes and RT-qPCR to examine the expression of early genes during osteogenic transdifferentiation in pre-myoblastic C2C12 cells, upon induction by rhBMP2 or rhBMP7. This effort resulted in the assessment of both previously known and novel genes which are commonly activated by both rhBMPs.

## Material and methods

### Cell Culture

The mouse pre-myoblastic and pre-osteoblastic C2C12 cell line was obtained from the American Type Culture Collection. These cells were maintained in DMEM (Gibco BRL, Gaithersburg, MD, USA) containing 10% FBS (HyClone^®^) and antibiotics (100 U/ml of penicillin-G and 100 mg/ml of streptomycin, GIBCO) at 37°C in a humidified atmosphere of 2.5% CO_2 _in air. For differentiation experiments, cells were seeded at a density of 2 × 10^4 ^cells/cm^2 ^and grown for 24 h. Subsequently, the medium was replaced by DMEM containing 5% FBS in the presence or absence of 200 ng/ml rhBMP2 or 200 ng/ml rhBMP7 (R&D Systems Inc., Minneapolis, MN). The activity of rhBMP2 and rhBMP7 was determined by the alkaline phosphatase activity assay in C2C12 cells.

### RNA isolation and DNA microarray hybridisation

C2C12 cultures were collected after 0, 4, 8, 12 and 24h of treatment with rhBMP2 or rhBMP7 for extraction of total RNA using the RNeasy kit (Qiagen, Hilden, Germany). Each cRNA was prepared as recommended in the Amersham CodelinkTM iExpress Assay Reagent Kit manual. Briefly, the samples containing 1.5 μg RNA of each culture treated for 12 h with each rhBMP (2 and 7) were mixed with 7.5 ml of bacterial mRNAs (10 pg/μl) and 1 μl of oligo dT to a final volume of 12 μl. Each reaction was incubated at 70°C for 10 min and kept on ice for 3 min. Subsequently, 2 μl of 10X First Strand Buffer, 4 μl of 5 mM dNTPs, 1 μl of RNase inhibitor and 1 μl of ArrayScript were added to each tube, to a final volume of 20 μl. Each reaction was incubated at 42°C for 2 h. After this period, each tube was placed on ice and 63 μl of water, 10 μl of 10X second strand buffer, 4 μl of 5 mM dNTPs, 2 μl of DNA polymerase and 1 μl of RNaseH were added to each tube. After incubating the reactions at 16°C for 2 h, each cDNA was purified and 20 μl of each purified cDNA was mixed with 12 μl biotin-NTP, 4 μl of 10X T7 reaction buffer and 4 μl of 10X enzyme mix reaction buffer, then incubated at 37°C for 14 h. After cRNA synthesis, each cRNA preparation was purified and 10 μg of each biotinylated cRNA was mixed with 5 μl of fragmentation buffer in a final volume of 25 μl, incubated at 94°C for 20 min and then kept on ice for 5 min. Subsequently, 25 μl of each reaction was transferred to another tube containing 78 μl of hybridisation buffer A, 130 μl of hybridisation buffer B and 27 μl of water; the reaction was incubated at 90°C for 5 min. Each tube was then placed on ice and applied to the Codelink DNA microarray containing around 36,000 genes. The subsequent stages of hybridisation, washing and analysis of each microarray were carried out as detailed in the Codelink Gene Expression System: Single-Assay Bioarray Hybridisation and Detection manual.

### DNA microarray analysis

The gene expression data were normalised using the SVR (Support Vector Regression) method [8], implemented in the GEDI toolbox [9]. More information on normalisation method is available at http://mariwork.iq.usp.br/gedi/. Briefly, SVR is a non-parametric regression, similar to the Loess method; however, it has greater accuracy than the Loess method, therefore, it is a more useful tool to identify differentially expressed genes. Identification of these genes was obtained using the ratio of normalised intensities between rhBMP (2 or 7) and control (untreated) samples, considering a fold change of ≥ 2.5. Induced and repressed genes in C2C12 cells under simultaneous treatment with rhBMP2 and rhBMP7 were classified according to their functions through the FatiGO program (http://bioinfo.cipf.es/babelomicswiki/tool:fatigo).

### Real Time RT-PCR

One microgram of total RNA was treated with 1 U of DNaseI at 37°C for 20 min and at 75°C for 10 min. The RT reaction was carried out at 50°C for 2 h in a 20 μl mix of 1X first strand buffer, 10 mM DTT, 2.5 mM of poly-dT primer (Amersham-Bioscience), 1 mM each of dNTPs, 20 U of RNAseOUT (Invitrogen) and 20 U of Superscript III (Invitrogen). The mixture was then incubated at 70°C for 15 min and incubated with 5 U of RNaseH at 37°C for 30 min and finally incubated at 75°C for 10 min. Samples of cDNA were diluted to 100 μl, and 3 μl aliquots of this cDNA solution were used for quantitative PCR using a Light-Cycler Real Time PCR System 7300 (Applied Biosystems). The PCR reaction was carried out in a 12 μl mix containing 6 μl of Sybr^®^Green Mastermix (Applied Biosystems), 3 μl of the cDNA solution and 3 μl of the appropriate primers in a concentration between 200 to 600 nm under the following conditions: 2 min at 50°C, 10 min at 95°C, followed by 40 cycles of 15 sec at 95°C and 1 min at 60°C. Expression values were calculated from threshold cycle at which an increase in reporter fluorescence above baseline signal could first be detected (C_T_). For data normalisation, three housekeeping genes were used, namely *mGAPDH*, *mHPRT *and *mHMBS*, using a calculated factor that was generated by the GeNorm program [10]. Control (untreated) and rhBMP2 and rhBMP7 treated groups were compared using a two-way ANOVA (Bonferroni test) and the GraphPad Prism 4 program. All primers used (Table 1) were validated beforehand according to the method described by Rasmussen [11].

**Table 1:** RT-PCR primers used in this study

Gene	Forward	Reverse
mG3PDH	GATGCCCCCATGTTTGTGAT	GGTCATGAGCCCTTCCACAAT
mHMBS	GCGGAAGAAAACGGCTCAA	TCCCGTGGTGGACATAGCA
mHPRT	GTCCCAGCGTCGTGATTAGC	TCATGACATCTCGAGCAAGTCTTT
Runx2	ACCGAGACCAACCGAGTCAT	CTCGGATCCCAAAAGAAGCTT
Dlx1	GTCTGTGCGCCGAAGTCAA	GCCCGGAAGAAGACCATTC
Dlx2	CAGCGGCCTCAACAATGTCT	ATTCGGATTTCAGGCTCAAGGT
Dlx5	GCCTCTCTAGGACTGACGCAAA	TGGTGACTGTGGCGAGTTACA
Id1	GATGGACTCCAGCCCTTCAG	TGGAGAGGGTGAGGCTCTGT
Id2	GGTGGACGACCCGATGAGT	GCGATCTGCAGGTCCAAGAT
Id3	GAAATCCTGCAGCGTGTCATAG	AAAGCTCCTCTTGTCCTTGGAGAT
Osx	TCCCCTTGTCGTCATGGTTAC	TTGAATTTGATCCCAGAGAAAGC
Hoxc8	ATGCCCAGCATACACTCTCTTGT	ATAAATACCAGAGAAGCACCGTGAA
Fkhr1	CCTTTGCCCCAGATGCCTAT	GGGATCAACCGGTGACATAATG
Glis1	AGAAGCCCAACAAGTGCATGTT	GGTCGCTGGAGTTGCTGAAG
Glis3	TGCACCTCTTCCATCTTCTCATT	TTCCCGTTCTTGTGCATATTCAT
Cfdp1	AAGAAGGGTACATTGAGCGGAAA	AGGAGCTCACATTGTCAATAGGATT
Lrp6	ACCAAGGTCCAGGCTCGAA	GCATCTTGTCGTACCATCTCCTTT
Dvl1	AAGAGTGACATGAGTGCCATTGTC	GAAGCCCTCCACGTGTGTGTA
Ecsit	AAGCTGTGGTTCACCCGATTC	GGGCAAAGACATCTGGTAGACAGT
Pkcd	GTGGTGTTGATTGACGATGATGTAG	ACCCCCATTGAGAAACTCCAT
Ltbp2	AGGAGCCAAGAGTTAAAGAAATCTAAAA	TTTCCCATAGGATAGATGTGCCTAA
Grn	CAATGCCCAATGCCATCTG	ACTTCACCTCCTTCACTGGGTATC
BMP1	CCATGTCTCTATTGTACGCGAGAA	AAGATGCCCCTGGAGAATGTG
Plod1	AAACGTGCCCACTATCGACAT	CGGACGACGAAGGCTAGATC
Postn	TTAGCATCTTCCTCAGCCTCCTT	GAGCATTTTTATCCCCAATCAGAA
Htra1	AGCTGAGACCTGGAGAATTTGTAGTT	TAGCGTCTGTCTGAATGTAGTCCAT
IGFBP-rP10	GCTGCAATCACGGAGTTTGTT	GTCTTCTCCGGGCTTTCTACAC

### Protein network analysis of gene products which were upregulated by treatment with rhBMP2/7

Accession numbers for genes validated by real-time RT-PCR were searched against the STRING database version 9 (http://string-db.org/) [12] for protein-protein interactions, using a STRING confidence score set to ≥ 0.7 (high confidence).

## Results

### Gene expression profiles during osteodifferentiation of C2C12 cells induced by rhBMP2 and rhBMP7 were constructed using DNA microarrays

In order to identify novel potential regulators of osteoblastic differentiation, we used DNA microarrays and RT-qPCR. Total RNA was isolated after 12 h of treatment with rhBMP2 or rhBMP7 for both DNA microarray and RT-qPCR experiments. In addition, total RNA was collected at 0, 4, 8 and 24h after stimulation with each of these BMPs for RT-qPCR analysis. Upon treatment of C2C12 cells with rhBMP2 or rhBMP7, the microarray analysis data resulted in 1,862 and 2,517 upregulated genes, and 1,335 and 1,918 downregulated genes, respectively, considering as significant a fold change of 2.5-fold or higher. Induced and repressed genes were intercepted, resulting in 1,318 induced genes and 704 repressed genes by both rhBMP2 and rhBMP7.

The differentially expressed genes regulated in common by rhBMP2 and rhBMP7 were classified according to their function, as predicted by the FatiGO Data Mining program (http://bioinfo.cipf.es/babelomicswiki/tool:fatigo), which classifies genes according to Gene Ontology (GO) criteria (cellular component, function and molecular biological process), thereby allowing the selection of 24 genes induced by both rhBMP2 and rhBMP7. These genes, which are related mainly to signal transduction, development, cell differentiation, osteodifferentiation and bone repair, are shown in Table 2, with the fold change for each gene also being shown.

**Table 2:** Genes selected by DNA microarray analysis during osteodifferentiation of C2C12 cells induced by both rhBMP2 and rhBMP7

Accession Number	Gene	Fold Change	
		rhBMP2/C	rhBMP7/C

NM_009820.1	Runt related transcription factor 2 (Runx2)	2.9	3.2
NM_010495.1	Inhibitor of DNA binding 1 (Id1)	20.4	31.7
NM_010496.2	Inhibitor of DNA binding 2 (Id2)	5.8	88.3
NM_008321.1	Inhibitor of DNA binding 3 (Id3)	3.6	4.09
NM_010053.1	Distal-less homeobox 1 (Dlx1)	6.5	8.0
NM_010054.1	Distal-less homeobox 2 (Dlx2)	3.2	5.6
NM_010056.2	Distal-less homeobox 5 (Dlx5)	4.2	4.5
NM_130458.1	Osterix transcription factor (Osx)	3.7	5.5
NM_010466.1	Homeobox C8 (Hoxc8)	2.5	4.2
NM_147221.1	GLIS family zinc finger 1 (Glis1)	176.8	131.3
NM_175459.3	GLIS family zinc finger 3 (Glis3)	35.5	33.3
AF114258.1	Forkhead protein 1 (FKHR1)	3.2	67.2
NM_011801.1	Craniofacial development protein 1 (Cfdp1)	4.3	46.3
NM_010091.3	Dvl1	4.3	160.2
NM_011103.1	Protein kinase C, delta (Prkcd)	2.5	11.2
NM_012029.1	Signalling Intermediate in Toll pathway-evolutionarily conserved (Ecsit)	2.9	12.8
NM_008175.2	Granulin (Grn)	3.1	89.0
NM_008514.1	Low density lipoprotein receptor-related protein 6 (Lrp6)	2.7	6.5
NM_013589.1	Latent transforming growth factor beta binding protein 2 (Ltbp2)	5.3	17.6
NM_178929.2	Kazal-type serine protease inhibitor domain 1 (IGFBP-rP10)	3.3	7.1
NM_015784.1	Periostin, osteoblast specific factor (Postn)	2.5	14.3
NM_019564.1	High Temperature requirement 1 (Htra1)	2.5	3.9
NM_009755.2	Bone morphogenetic protein 1 (Bmp1)	4.1	55.4
NM_011122.1	Procollagen-lysine, 2-oxoglutarate 5-dioxygenase 1 (Plod1)	37.9	45.9

Differential gene expression was validated by RT-qPCR. The results represent the mean values of three independent experiments, each of which was performed in triplicate. The relative expression of each gene was calculated as described in the Materials and Methods.

**Figure 1:**
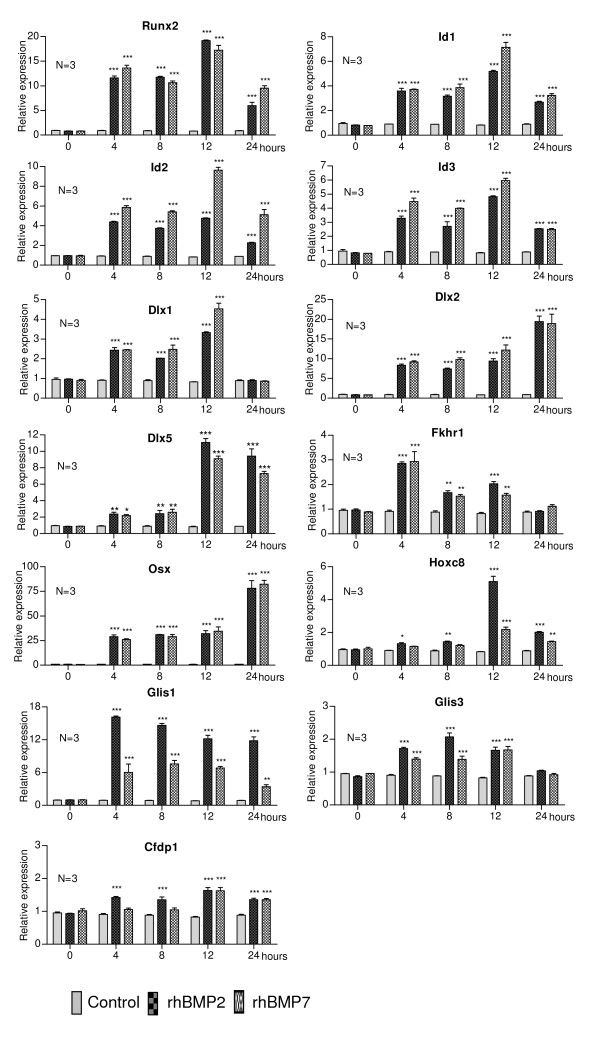
**Relative expression levels of genes related to transcription in C2C12 cells treated with rhBMP2 and rhBMP7**. Cells were seeded and grown for 24 h at which point the medium was replaced by DMEM containing 5% FCS in the presence or absence of rhBMP2 (200 ng/ml) or rhBMP7 (200 ng/ml). RNA was isolated 0, 4, 8, 12 and 24h after stimulation. Subsequently, expression levels were measured by quantitative real time RT-PCR. The asterisks correspond to the statistical analysis (two-way ANOVA, Bonferroni test) between control and treatment (*p < 0.05; **p < 0.01; ***p < 0.001).

Figure 1 shows the expression profile of 13 genes involved in the transcriptional regulation of several genes, some of which are already known to be involved in the regulation of different cellular processes, while others have not yet been characterised, namely: *Runx2, Dlx1, Dlx2, Dlx5, Id1, Id2, Id3, Fkhr1, Osx, Hoxc8, Glis1, Glis3 *and *Cfdp1*. *Id1*, *Id2 *and *Id3 *correspond to proteins which bind to transcription factors, modifying their ability to bind DNA and affecting the expression of one or more genes. *Cfdp1 *could participate in the complex of proteins that modify the chromatin state, thus regulating the transcription of several genes. All of the other genes presented in Figure 1 code for transcription factors. The first gene is the well-known *Runx2*, which was induced to similar levels by rhBMP2 and rhBMP7. *Runx2 *was upregulated four hours after treatment, and the highest level of expression appeared 12 h after treatment, declining at 24 h, but remaining significantly higher than the control. This same expression profile was observed for *Id1, Id2 *and *Id3*, but, in the case of *Id2*, treatment with rhBMP7 caused a higher level of expression than rhBMP2 at 4, 8, 12 and 24 h; for *Id1 *and *Id3*, there was also a difference in induction over these time periods, but it was lower than that achieved by *Id2*. *Dlx1 *and *Dlx2 *were induced 4 h after stimulation with both rhBMPs. *Dlx1 *reached its maximum expression 12 h after treatment, then decreased to basal levels by 24 h. During this period of time, *Dlx2 *reached its highest expression level. *Dlx5 *was similarly upregulated by both rhBMPs, with a slight increase between 4-8 h and a further increase (5 to 6-fold) at 12 and 24 h. Fkhr1 was induced, displayed its maximum expression level after 4 h and decreased thereafter to basal levels at 24 h. The expression of *Osx *began to increase 4 h after treatment and gradually increased until reaching its maximum level after 24 h; expression levels were similar upon treatment with both proteins. *Hoxc8 *expression slightly increased after 4 and 8 h, reached its maximum level at 12 h and decreased at 24 h after treatment with rhBMP2, while treatment with rhBMP7 caused increased expression only at 12 and 24 h. Another difference was that, after 12 h of treatment, rhBMP2 caused greater expression of Hoxc8 than rhBMP7. *Glis1 *was upregulated at 4 h, and remained at a constant level for up to 12h, decreasing at 24 h, but, in this case, there was a clearly greater increased expression when C2C12 cells were treated with rhBMP2 than with rhBMP7. *Glis3 *was induced after 4 h of treatment, remained constant for up to 12 h and decreased at 24h, but, unlike *Glis1*, the level of expression achieved by *Glis3 *was very similar in cells treated with rhBMP2 or rhBMP7.

The differential expression of the *Cfdp1 *gene is shown in Figure 1. This gene was induced by rhBMP2 at 4 h, was maintained at a constant expression level for up to 8 h, slightly increased at 12 h and decreased again at 24 h to similar levels as those detected at 4 or 8 h. On the other hand, the expression of *Cfdp1 *only significantly increased 12 h after treatment with rhBMP7 but by 24 h was expressed at a higher level when compared to control.

**Figure 2:**
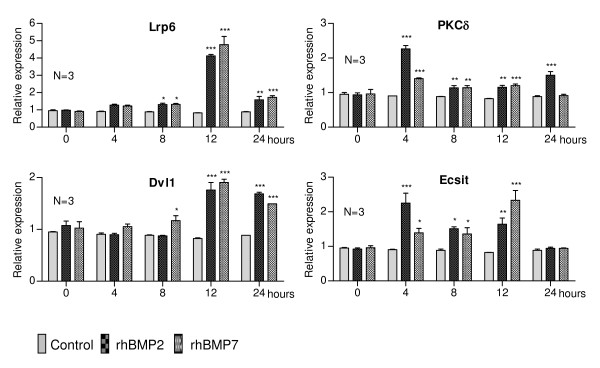
**Relative expression levels of genes related to cell signalling in C2C12 cells treated with rhBMP2 and rhBMP7**. Cells were seeded and grown for 24 h at which point the medium was replaced by DMEM containing 5% FCS in the presence or absence of rhBMP2 (200 ng/ml) or rhBMP7 (200 ng/ml). RNA was isolated 0, 4, 8, 12 and 24h after stimulation. Subsequently, expression levels were measured by quantitative real time RT-PCR. The asterisks correspond to the statistical analysis (two-way ANOVA, Bonferroni test) between control and treatment (*p < 0.05; **p < 0.01; ***p < 0.001).

Figure 2 shows the expression analysis of g*enes related to cell signalling: Lrp6*, *Dvl1*, *Ecsit *and *PKCδ*. The expression of *Lrp6 *slightly increased after 4 and 8 h of treatment with rhBMPs, then reached maximum expression levels at 12 h and decreased by 24h. Treatment with rhBMP7 caused a slight increase in *Dvl1 *expression at 8 h while rhBMP2 did not generate such a response. Coincidentally, maximum expression was found at 12 h in cells treated with both recombinant proteins, with slightly decreasing levels until up to 24h. The expression of *Ecsit *increased 4 h after stimulation, slightly decreased at 8 h increased again at 12 h and decreased to basal levels at 24 h. In the case of *PKCδ*, treatment with rhBMP2 for 4 h caused greater expression when compared to treatment with rhBMP7. However, at 8 and 12 h, the levels achieved with both treatments were very similar. Finally, at 24 h, greater expression was found in cells treated with rhBMP2, when compared to the control and to treatment with rhBMP7, but the expression of this gene decreased at this time point.

**Figure 3:**
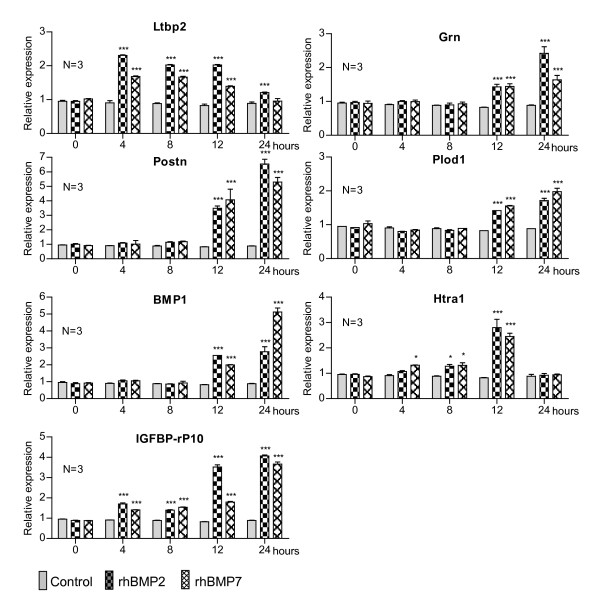
**Relative expression levels of genes related to the extracellular matrix in C2C12 cells treated with rhBMP2 and rhBMP7**. Cells were seeded and grown for 24 h at which point the medium was replaced by DMEM containing 5% FCS in the presence or absence of rhBMP2 (200 ng/ml) or rhBMP7 (200 ng/ml). RNA was isolated 0, 4, 8, 12 and 24h after stimulation. Subsequently, expression levels were measured by quantitative real time RT-PCR. The asterisks correspond to the statistical analysis (two-way ANOVA, Bonferroni test) between control and treatment (*p < 0.05; **p < 0.01; ***p < 0.001).

Upon analysis of the DNA microarrays, seven genes related to the extracellular matrix were selected to be examined by RT-qPCR, namely *Ltbp2*, *Grn*, *Postn*, *Plod1*, *BMP1*, *Htra1 *and *IGFBP-rP10; *the results are shown in Figure 3. The expression of *Ltbp2 *increased upon BMP stimulation 4 h after treatment, maintained a constant level of expression for up to 12 h, but decreased after 24 h. As can be observed, slightly higher expression was found in cells were treated with rhBMP2 compared to cells treated with rhBMP7. A similar expression profile was found for *Grn, Postn, Plod1 *and *BMP1*. Coincidentally, increased expression of these four genes occurred after 12 h of stimulation with rhBMP2 and rhBMP7 and continued to increase at 24 h. Upon incubation with both rhBMPs, the expression of *Htra1 *began to gradually increase at 4 h, was maintained at this level at 8 h, reached a peak at 12 h and fell to basal levels at 24 h. As can be seen in Figure 3, the expression of *IGFBP-rP10 *was induced at 4 h after treatment and remained at a constant level at 8 h and increased again at 12 h, with higher levels in cells incubated with rhBMP2 than with rhBMP7. At 24 h, the level of expression induced by rhBMP2 was very similar to that obtained at 12 h, or remained virtually the same, except for the fact that the expression induced by rhBMP7 at 24 h was about twice the level seen at 12 h.

## Discussion

Our DNA microarray analysis refers to treatment of pre-myoblastic and pre-osteoblastic C2C12 cells with BMP2 and BMP7 for a period of 12 h. The choice of this period relies on the fact that expression of the genes which induce C2C12 cells to differentiate into osteoblast-like cells is found upon treatment with rhBMP2 for 8 and 16 h [13]. The cut-off of 2.5-fold change was chosen due to the fact that Runx2, which is widely known to be activated by both rhBMPs during osteodifferentiation [14], presented a fold change of 2.94 and 3.17 upon treatment with rhBMP2 and rhBMP7, respectively (Table 2). Although downregulated genes are also of interest, they were not selected due to the fact that, in C2C12 cells, most of these genes would probably be more directly involved in myogenesis rather than in osteogenesis, but this does not rule out the interest in their analysis in future research. Therefore, our main interest was focused on genes which are upregulated by both rhBMPs and that could be involved in osteodifferentiation. Particularly, we focused on genes which are induced at the very beginning of osteoblast differentiation, which encompasses the first 24 h following BMP receptor activation, a period in which the key process for commitment to the osteoblast lineage occurs in precursor cells.

The genes selected from the microarray analysis were classified according to their function, 13 of which are involved in transcription, four in cell signalling and seven are related to the extracellular matrix. The mRNA levels of their corresponding genes, determined by qRT-PCR, were analysed at different time points (0, 4, 8, 12 and 24h) following treatment with both BMPs, each displaying its own expression profile, which was compared to that of the other.

### Transcription factors and related proteins

Most biological processes, including osteoblastic differentiation, are dependent upon transcriptional activation of several effector genes. The signal generated upon interaction between BMPs and their receptors causes activation and repression of a number of genes coding for transcription factors and, also, for proteins, which bind to these factors, thereby positively or negatively modifying their function. The expression profile of 13 genes related to transcription was examined by RT-qPCR (Figure 1).

#### Runx2

**Figure 4:**
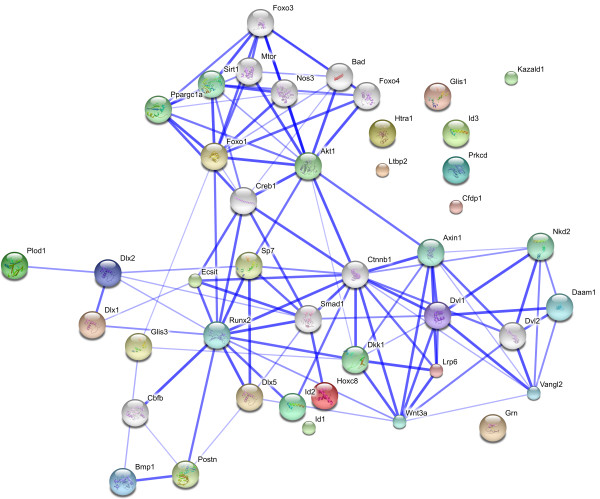
**Network of upregulated genes in C2C12 cells treated with rhBMP2 and rhBMP7 using the STRING 9.0 pathway analyzer**. STRING 9.0 is a database of known and predicted protein interactions. The interactions may be physically direct or indirect (functional). The list of protein names are inserted as input and the organism to be analyzed is selected in a user-friendly web-browser interface. The network, composed of the uploaded proteins, is constructed based on four different sources: genomic context, high-throughput experiments, co-expression and scientific reports (http://string-db.org/). The upregulated genes found in each group (C2C12 cells treated with rhBMP7 or rhBMP2) were matched and classified according to a pre-described interaction (activation, repression, or predicted interaction). The circles represent the genes and the blue edges represent the interaction between them. The edges are based on proteins being co-mentioned in scientific publications as well as their association in curated databases. The edge thickness is directly proportional to the degree of evidence available, with lack of evidence for interactions in the literature being represented as no edges among the genes. Runx2: Runt Related Transcription factor 2; Dlx1/2/5: Distal-less homeobox 1/2 and 5;Id1/2/3: Inhibitor of DNA binding 1/2 and 3; Sp7, an alternative name for Osterix: Transcription factor Sp7; Hoxc8: Homeobox protein Hox-C8; Fkhr1: Forkhead protein FKHR1; Glis1/3: Gli homologous protein 1; Cfdp1: Craniofacial development protein 1; Lrp6: Low-density lipoprotein receptor-related protein 6; Segment polarity protein dishevelled homolog DVL-1; Ecsit: Evolutionarily conserved signaling intermediate in the Toll pathway, mitochondrial; PKCd: Protein kinase C, delta type; Ltbp2: Latent-transforming growth factor beta-binding protein 2; Grn: Granulin; BMP1: Bone Morphogenetic Protein 1; Plod1: Procollagen-lysine,2-oxoglutarate 5-dioxygenase 1; Postn: Periostin; Htra1: Serine protease HTRA1; Kazald1: Kazal-type serine protease inhibitor domain-containing protein 1 (IGFBP-rP10); Wnt3a: Protein Wnt-3a; Dkk1: Dickkopf-related protein 1 and Ctnn1b: Catenin beta-1.

As expected, *Runx2 *was induced upon 4 h of treatment with both rhBMPs. This gene is at the end of the Smad protein pathway, and, as recently found [7], the MAPK pathway. As such, its product is a very important transcription factor for both chondrogenesis and osteogenesis during the development of the skeleton [14]. In addition to BMPs, *Runx2 *is also induced by TGFβ and FGF2, which are also known to be involved in bone formation [15]. *Runx2*^*-/- *^knock-out mice show a complete lack of functional osteoblasts, and are devoid of mineralised bone or hypertrophic cartilage [16]. Moreover, mutations in this gene result in cleidocranial dysplasia, which is characterised by bone alterations [17]. BMP2 and Runx2 act synergistically, stimulating osteodifferentiation both *in vitro *and *in vivo *[18]. Indeed, Runx2 phosphorylation, through MAPK activation by FGF2, seems to be important for the induction of osteocalcin expression [19]. Therefore, the importance of Runx2 in osteoblast differentiation and bone formation is clear: it represents a hub gene in bone formation by regulating osteoblast genes (Figure 4), along with the fact that this gene is differentially expressed in C2C12 cells treated with rhBMP2 and rhBMP7 (Figure 1), conferring greater confidence with respect to the other genes selected in this study.

#### Id1, Id2 and Id3

The *Id1, Id2 *and *Id3 *genes were induced by treatment of C2C12 cells with both rhBMPs (Figure 1). *Id *genes encode proteins called ID (inhibitor of DNA binding), a group of proteins which belong to the basic helix-loop-helix (bHLH) family, but lack the basic region, which is important in DNA binding and transcriptional activation. Evidence is available showing that Id1 forms heterodimers with E12/E47 and prevents MyoD from forming complexes with E12/E47 in myoblasts [20,21]. The Id proteins are targets for BMP family members, particularly for BMP2 [22,23]. Interestingly, a Smad phosphatase, named C-terminal domain phosphatase (SCP1) inhibited the activation of Id1, Id2 and Id3, impairing osteoblastic differentiation in C2C12 cells [24]. These effects on Id genes expression were only described for cells treated with BMP2, as no report is yet available for BMP7-treated cells. Together, the results we present here and those of others [21] indicate that these Id genes are, in fact, activated during osteoblastic differentiation upon treatment with both rhBMP2 and rhBMP7.

#### Dlx1, Dlx2 and Dlx5

Treatment with rhBMP2 and rhBMP7 induced the homeobox gene family related to the distal-less (Dll) gene of Drosophila (*Dlx1, Dlx2 *and *Dlx5 *genes, Figure 1) [25]. The *Dlx *genes control the expression of various genes involved in the development of invertebrates and vertebrates. In mammals, they are expressed in the nervous system derived from the neural crest and brachial arches, participating in the development of the brain, craniofacial structures and the axial and appendicular skeleton [25]. In view of these characteristics, the Dlx1, Dlx2 and Dlx5 transcription factors may be very important during osteoblastic differentiation. Particularly, evidence is available indicating that *Dlx5 *is a direct target for BMPs. During osteogenic transdifferentiation of C2C12 cells, it has been demonstrated that Dlx5 regulates the expression of *Runx2 *in cells treated with rhBMP2, but not with TGFβ1 [26] (interaction between Runx2 and Dlx5, as shown in Figure 4). This, together with the ability of BMP2 to induce the expression of alkaline phosphatase (*ALP*) and *Osx *in Runx2^-/- ^knock-out mouse cells, could represent an additional pathway by which Dlx5 modulates the expression of *Runx2 *and *Osx*. Dlx5, but not Runx2, is essential for the expression of *Osx *in cells treated with BMP2 [15], indicating that Dlx5 regulates the expression of *Osx *independently of Runx2. These data are consistent with the *Dlx5 *gene expression profile obtained here for both rhBMP treatments, so it is likely that Dlx1 and Dlx2 could also participate in this or similar mechanisms.

#### Osx

Osx, a zinc finger-containing transcription factor, was the gene with the greatest relative induction level upon treatment of C2C12 cells with rhBMPs. This gene is expressed in all bones, and *Osx^-/- ^*knock-out mice lack mineralised bone [27]. It has been suggested that Osx acts in the terminal differentiation of osteoblasts to operate the distinction between chondrogenic and osteogenic pathways while Runx2 acts upstream, in the commitment of mesenchymal cells to chondrocytes or osteoblasts [28]. Osx regulates the expression of important genes in bone, such as those coding for bone sialoprotein (*BSP*), *osteocalcin*, *osteonectin *and *osteopontin *[29]. As mentioned above, Dlx5 regulates the expression of *Osx *(also known as Sp7, node in Figure 4). Interestingly, confirming data from the literature on the expression profile of *Dlx2 *and *Dlx5*, it may be noted that the *Osx *transcript significantly increased between 12 and 24 h, which may be related to the increase in *Dlx2 *and *Dlx5 *expression during the same period of time, revealing that although *Osx *first requires Dlx5, later on, Dlx2 could also be important for resuming osteodifferentiation (Figure 1).

#### Fkhr1

The Fkhr1 transcription factor, which we found to be differentially expressed in C2C12 cells, is able to stimulate alkaline phosphatase gene expression, increasing its activity. This protein and the family of factors to which it belongs are involved in regulating processes such as the cell cycle, apoptosis, glucose metabolism, cell differentiation and embryogenesis [30]. Recently, a study demonstrated that Fkhr1 is involved in osteoblastic differentiation of MC3T3-E1 cells, since Fkhr1 knockdown cells failed to perform matrix mineralisation, and immunoprecipitation assays suggest an interaction between Fkhr1 and the RunX2 promoter [31]. Moreover, increased *Fkhr1 *expression and ALP activity were detected six days after treatment of hMSC (human marrow cells) with BMP2 [32]. We found that the expression fell to basal levels 24 h after treatment in both BMP2 and BMP7-treated cells (Figure 1), while the ALP activity began to gradually increase after 48 h of treatment (data not shown). A few explanations for this may be envisaged, namely: a) *ALP *expression is activated by other transcription factors, such as Runx2 and Dlx5; b) the decrease in the level of *Fkhr1 *transcripts does not mean that the same occurs at the protein level; 3) the *Fkhr1 *transcript level could increase again 24 h after stimulation.

#### Hoxc8

*Hoxc8 *belongs to the family of conserved Hox genes, which are expressed in the neural tube, cartilage and bone [33]. Hoxc8 binds to the osteopontin (*Spp1*) and osteoprogeterin (*OPG*) promoters, acting as a transcriptional repressor. We showed that *Hoxc8 *was induced by rhBMP2 between 4 and 24 h, and by rhBMP7 between 12 and 24 h (Figure 1). Despite the role of *Hoxc8 *in the inhibition of osteoblastogenesis, evidenced by stimulation of Smad6 expression in BMP2-treated cells [34], its expression could be explained by a negative feedback loop, regulating the activated Smads, similar to what occurs in *in vivo *bone metabolism. Recently, it has been reported that BMP2 regulates the expression of miR-199a* microRNA, which, in turn, downregulates the expression of Smad1. Therefore, the increased expression of *Hoxc8*, caused by BMPs, may be related an inhibition in Smad1 function (Figure 4) additionally caused by miRNA-199a* [35]. It is interesting to note that a high fold-induction of *Hoxc8 *was obtained in rhBMP2-treated cells, whereas only slightly increased expression was observed for rhBMP7-treated cells, when compared to the control. This suggests that the difference in receptor binding between each BMP implies not only common intracellular messengers, but also activation of different intracellular proteins which may be responsible for this difference in expression levels.

#### Glis1 and Glis3

rhBMP2 and rhBMP7 also induced the *Glis1 *and *Glis3 *(Gli similar) genes (Figure 1), both of which code for transcription factors belonging to the family of Krüppel-like zinc finger proteins [36]. Glis1 and Glis3 contain five Cys2-His2-type zinc-finger motifs which exhibit high homology with those of Gli. However, Glis and Gli proteins exhibit low sequence homology outside their zinc-finger domain. Deletion analysis demonstrated that Glis proteins contain both repressor and activator domains, suggesting that they may function as positive, as well as negative, regulators of gene transcription, and evidence is available suggesting that Glis3 participates in various stages of organogenesis [15]. The expression of Glis1 has been detected during embryogenesis in craniofacial regions, brachial arches and kidney somites [36]. Glis3 has recently been shown to operate synergistically with BMP2 and Shh in osteoblastic differentiation of C3H10T1/2 mesenchymal cells, inducing the expression of *FGF18 *[37], indicating that this factor may be important during osteodifferentiation. Here, we show that *Glis1 *expression increased upon treatment with rhBMPs between 4 and 24 h, (Figure 1), resulting in a greater transcript level increase in cells treated with rhBMP2. To date, no evidence is available relating Glis1 with osteodifferentiation induced by BMPs, but its role could possibly be similar to that of Glis3, due to the high homology between these genes. Undoubtedly, in view of the scarce knowledge on the potential role of these transcription factors in osteoblastic differentiation, future studies should be directed to clarify their importance in this cellular process.

#### Cfdp1

*Cfdp1 *expression was induced earlier by rhBMP2 (4-24 h) when compared to rhBMP7 (12-24 h) (Figure 1). Cfdp1 has been detected in some tissues, particularly in teeth and bones. This protein contains a domain called BCNT-c (Bucentaur), which displays homology to one of the proteins constituting the yeast Swr1 complex, which modifies the chromatin state, thereby transcriptionally regulating several genes. *Cfdp1 *was cloned from a mouse library, and the equivalent region in the human genome was found to be associated with craniofacial syndromes. The specific function of Cfdp1 is unknown, but it is presumed to be important during embryogenesis [38]. Evidence is available indicating that Cfdp1 participates in tooth development [39] and could play a similar function during bone formation. Further investigation is necessary to establish the actual role of Cfdp1 in osteogenesis.

### Genes involved in cellular signalling

BMP-activated cell signalling involves the Smad proteins [40]. Interestingly, our analysis of microarrays generated four candidate genes which participate in other pathways, such as *Wnt *(canonical and non-canonical) and *Toll*, indicating that these pathways could interact and take part during osteoblastic differentiation. Figure 2 shows a similar expression profile for the *Lrp6 *and *Dvl1 *genes upon treatment with rhBMPs. These genes participate in canonical Wnt signalling [41], with evidence showing their relationship with osteogenesis [42,43]. *Lrp6^-/- ^*knock-out mice display reduced vertebral trabecular bone volume (TBV), demonstrating the importance of LRP6 in bone formation [44]. Moreover, it has been shown that this co-receptor participates in somitogenesis and osteogenesis [45]. Dvl1 participates, along with other proteins, in the stabilisation of β-catenin, thus inhibiting the activity of GSK3β kinase [46]. Some data suggest that Dvl1 and? other components of Wnt signalling are activated during bone regeneration and that, particularly, the Dvl isoforms (1, 2 and 3) could be important in the proliferation and differentiation of chondrocytes [41]. The increased expression of *Lrp6 *and *Dvl1*, observed here, could mean that treatment of C2C12 cells with rhBMPs leads to increased expression of genes related to the signalling, thereby intensifying the effect initially induced by BMPs. In this respect, using C2C12 and C3H10T1/2 cells, a synergy has been shown between β-catenin and BMP2 to promote osteodifferentiation [47]. Also, it is possible that β-catenin, along with TCF1, may increase *Runx2 *expression [48].

#### Ecsit

Increased expression of *Ecsit *was observed between 4 and 12 h of treating C2C12 cells with BMPs (Figure 2). Ecsit participates in the Toll pathway, which regulates pro-inflammatory genes. Ecsit also participates in BMP/Smad signalling, interacting with Smad1 and Smad4 and activating the expression of *Tlx2 *and other genes [49]. During embryogenesis, the Toll and BMP/Smad pathways interact for dorsoventral axis formation. On the other hand, Ecsit^-/- ^knock-out mice displayed alterations in epiblast and mesoderm formation, dying at embryonic day 7.5 (E7.5) [50]. The exact mechanism by which this protein could be involved in osteoblastic differentiation is unknown, requiring further analysis, but its interaction with the Smad1/4 proteins, and possibly with RunX2 (Figure 4), which are both known to be involved in osteodifferentiation, suggests that Ecsit is part of this cellular process, making it likely that the Toll pathway could also be involved in osteoblast differentiation.

#### PKCδ

Another gene associated with cell signalling, which we found to be induced by BMPs and validated by RT-qPCR, is *PKCδ*. The enzyme encoded by this gene belongs to the protein kinase C (PKC) family, which consists of 11 members [51]. Recently, using ST2 cells, it has been shown that PKCδ activation is induced by Wnt3a through the canonical pathway, and also by the non-canonical Wnt pathway through Wnt7b in ST2 and C3H10T1/2 cells. These results indicate the involvement of PKCδ during osteodifferentiation in these cells, which could indicate that Wnt7b and PKCδ regulate the onset of *Osx *expression [52]. Another study showed activation of the PKC pathway in MC3T3 and C2C12 cells treated with FGF2, with PKCδ being one of the key isoforms involved in FGF2-stimulated *Runx2 *expression [15].

### Proteins associated with the extracellular matrix

Osteoblasts secrete an extracellular matrix (ECM) formed by various proteins, some of which directly participate in mineralisation of the osteomatrix [19]. For this reason, seven genes representing secreted proteins, with the exception of Plod1, an intracellular enzyme, were analysed. Figure 3 shows that the expression of *Ltbp2 *increased upon treatment with rhBMPs. This protein is part of the latent TGFβ complex, placing it in the ECM. TGFβ is activated upon removal from this complex [53]. A possible relationship between Ltbp2, TGFβ and rhBMPs induced transdifferentiation of C2C12 cells, which may represent, in part, what occurs in bone metabolism, since TGFβ participates in osteoclastogenesis, regulating the expression of *osteoprotegerin (OPG) *and the receptor activator of the *NF-κB ligand (RANKL) *in osteoblasts and, also, the receptor activator of *NF-κB (RANK) *in osteoclasts. Therefore, it is important that TGFβ be released from this latent complex in order to exert its effect [54].

#### Grn, Postn and Plod1

Coincidentally, our results show that *Grn, Postn, Plod1 *and *BMP1 *were induced between 12 and 24 h after treatment with rhBMPs. Grn (granulins) are proteins involved in inflammation, repair and remodeling of injured tissues. Although the function of Grn in osteodifferentiation is unknown, it has an osteoclastic origin [55]. Possibly, Grn could participate in bone remodeling, but, obviously, further studies should be carried out to investigate, in more detail, the functions of these proteins in bone tissue. Postn was isolated from osteoblasts as a specific factor, shown to be preferentially expressed in the periosteum. The function of Postn is related to recruitment, adhesion and extension of osteoblasts [13]. Interestingly, the expression of Postn began to increase after 12 h and progressively increased up to 24 h, indicating that the cells were beginning to display characteristics found in differentiated osteoblasts. Plod1 is not secreted, as it is an intracellular enzyme involved in lysine hydroxylation of type I collagen, the main protein present in bone. In agreement with our results, increased expression of Plod1 was obtained during differentiation of bone marrow stromal cells (BMSCs) and normal skin fibroblasts (NSFs) [56]. Therefore, increased expression of Plod1 during osteoblastic differentiation implicates a stabilisation of collagen type I, which may indicate the beginning of extracellular matrix formation in osteoblasts. BMP1 is an important metalloproteinase in morphogenetic processes in several species. During ECM formation, BMP1 performs the processing of precursor molecules, rendering them into functional ECM components. The substrates of BMP1 include pro-collagens, proteoglycan precursors and proteins associated with ECM mineralisation of bone and teeth. Some substrates are members of the latent complex of TGFβ family members, such as TGFβ1, BMP2 and BMP4 [57]. Recent evidence has shown that BMP1 cleaves LTBP molecules, releasing the latent complex of TGFβ1-LAP from the ECM; subsequently, the LAP protein (latency-associated peptide) is cleaved by another protease, resulting in the activation of TGFβ [58]. Our results show the same expression profile for *LTBP2 *and *BMP1 *upon induction by rhBMP2 and rhBMP7, associating them with ECM formation and osteoclastogenesis and indicating that both proteins participate in the regulation of TGFβ1, which is known to be important in such processes.

#### Htra1

Htra1 is a family of serine peptidases. It has recently been demonstrated that over-expression of *Htra1 *during osteoblastic differentiation of 2T3 cells causes inhibition of ECM mineralisation, suggesting that this may be due to one of three different causes: a) Htra1 modulates the expression of specific osteoblast genes; b) Htra1 alters the activity TGFβ/BMP pathway; or c) Htra1 cleaves proteins that regulate ECM differentiation and/or mineralisation [59]. Inhibition of ECM mineralisation in these cells by Htra1 could represent a mechanism to control the speed at which the mineralisation process occurs, but more evidence is required to confirm this hypothesis. The possible effect of Htra1 in the ECM mineralisation of C2C12 and/or other pre-osteoblastic cells should be further examined.

#### IGFBP-rP10

IGFBP-rP10 is a member of the insulin growth factor binding protein (IGFBP) family. These proteins are involved in the growth and maturation of various tissues through their association with ECM proteins, mediating the access, migration and cellular chemotaxis. IGFBPs bind to integrins, possibly modulating the TGFβ and Wnt pathways. According to our results, rhBMP2 is able to induce the expression of *IGFBP-rP10 *in different cell lines, in addition to C2C12 cells, rendering it very likely that IGFBP-rP10 actively participates in the differentiation and proliferation of osteoblasts during bone formation and bone regeneration [60]. The mechanism by which this protein is induced by BMP2 and BMP7 and how it is involved in these cellular processes represents an interesting challenge which should be faced in the future.

In summary, using DNA microarrays, we have described the pattern of expression of genes which are induced during transdifferentiation of pre-myoblastic C2C12 cells during 24 h of treatment with rhBMP2 and rhBMP7. A total of 1,318 genes were found to be induced by both rhBMPs which could be involved in osteoblastic differentiation, twenty-four of which were confirmed through RT-qPCR after 4, 8, 12 and 24h of treatment, resulting in 13 transcription-related genes (*Runx2, Dlx1, Dlx2, Dlx5, Id1, Id2, Id3, Fkhr1, Osx, Hoxc8, Glis1, Glis3 *and *Cfdp1*) which were induced between 4 and 24 h after treatment. Four of the genes identified (*Lrp6, Dvl1, Ecsit *and *PKCδ*) are implicated in cell signalling, displaying a more heterogeneous pattern of expression, and seven are ECM-related genes (*Ltbp2, Grn, Postn, Plod1, BMP1, Htra1 *and *IGFBP-rP10*), which were preferentially activated after 12 h of treatment. Our results revealed some genes which were already known to be activated by BMP2. However, we found that many of them were also upregulated by treatment with rhBMP7, thus representing a novel subset of genes related with BMP7-induced differentiation of precursor cells into osteoblasts: *Hoxc8, Glis1, Glis3, Ecsit, PKCd, LrP6, Dvl1, Grn, BMP1, Ltbp2, Plod1, Htra1 *and *IGFBP-rP10*. Fifteen genes were upregulated in the early period (during the first 12 h) while nine genes were upregulated at a later period (between 12 and 24 h). Among the downregulated genes, MyoD showed a decreased abundance in mRNA relative expression (data not shown), which is explained by the fact that, in the absence of BMPs, this muscular satellite cell line would be programmed to differentiate into myoblasts [20]. Currently, we do not know whether the increased expression of genes at later stages was merely due to the indirect downstream effects of BMP/SMAD signalling as a result of earlier gene (for example, Ids) expression changes. Subsequent studies may elucidate whether the products of these upregulated early genes are responsible for inducing the expression of later genes.

The network analysis of the upregulated genes showed a high connectivity for most of them, with some hubs being distinguished by having six interactions or more, namely a) RunX2, a well-known player in osteoblastogenesis; b) Smad 1, an intracellular transducer of BMP proteins; c) Akt1, a kinase recently found to induce Osx transcription, showing evidence for crosstalk between BMP- Smads and PI 3-kinase/Akt signalling [61]; and d) the Dvl1/Ctnnb1 (β-catenin 1) axis, both of which belong to the Wnt pathway. β-catenin 1 is described to be activated upon BMP2 treatment in C3H10T1/2 cells, playing a role in the Wnt-independent pathway mechanism of bone differentiation [62]. These data suggest that the abovementioned genes, despite belonging to distinct pathways, are related to each other in BMP-driven osteoblastogenesis.

It is noteworthy that the results presented here apply to this model of C2C12 osteoblastic differentiation, which may or may not be identical to that of other types of osteoblastic progenitors, due to the availability of BMP receptors on their surface and on how capable they are of triggering intracellular downstream pathways.

BMP2 and BMP7 both signal through the ALK2 and ALK3 receptors; however, BMP7 predominantly signals via the ALK2 receptor. This alternative signalling mechanism might account for some of the differences in the level of gene expression reported following BMP2 and BMP7 stimulation. The fact that there are differences in the levels of gene expression induced by BMP2 and BMP7 was proven, *in vivo*, to be due to different biological steps of activation of differentiation. Thus, while BMP2 plays a role in the early stages of osteoblastic differentiation, BMP7 participation is more delayed, sustaining osteoblast maturation, rather than triggering the initial differentiation signal [63]. Another possibility is the existence of unknown receptors or other pathways directly modulating the action of each BMP, as already described for MAPK and/or indirectly, as described by the Wnt pathway (the node representing the Wnt3a protein was found to interact with Lrp6, Ctnnb1, Dvl1, Dkk1 and Dlx5 in Figure 4) [64]. Therefore, subsequent studies should address whether BMP7 signalling through the ALK2 receptor may generate differences in gene expression for Id2 and Dvl1 (and other genes), as compared with BMP2 signalling.

Our findings are relevant to better understanding the molecular basis for BMP-induced osteogenesis during development and the bone healing processes; however, further studies are still required to investigate the intricate network of pathways triggered by these cytokines.

## Competing interests

The authors declare that they have no competing interests.

## Authors' contributions

JCBV has made substantial contributions to the conception and design of the study. AF has made contributions to the analysis and interpretation of mathematical results. EH has made contributions to the analysis and interpretation of biological data. JMG and MCS have discussed the biological results. MCS has directed the work. JCBV, AF, EH, MJG and MCS critically revised the manuscript for important intellectual content. All authors read and approved the final manuscript.
